# Longitudinal changes in fasting plasma glucose are associated with risk of cancer mortality: A Chinese cohort study

**DOI:** 10.1002/cam4.4070

**Published:** 2021-06-21

**Authors:** Mengyin Wu, Jieming Lu, Zongming Yang, Peng Shen, Zhebin Yu, Mengling Tang, Mingjuan Jin, Hongbo Lin, Kun Chen, Jianbing Wang

**Affiliations:** ^1^ Department of Epidemiology and Biostatistics School of Public Health Zhejiang University Hangzhou China; ^2^ Department of Chronic Disease and Health Promotion Yinzhou District Center for Disease Control and Prevention Ningbo China; ^3^ Cancer Institute The Second Affiliated Hospital Zhejiang University School of Medicine Hangzhou China; ^4^ Department of Epidemiology and Biostatistics The Children's Hospital National Clinical Research Center for Child Health Zhejiang University School of Medicine Hangzhou China

**Keywords:** cancer mortality, cohort study, fasting plasma glucose, longitudinal change, variability

## Abstract

**Background:**

Numerous studies have suggested that fasting plasma glucose (FPG) was associated with the risk of mortality. However, relationship on longitudinal changes of FPG with the risk of mortality remained inconsistent.

**Methods:**

We examined the association of FPG at baseline and its longitudinal changes with risk of mortality based on a cohort study in Yinzhou, China, during 2010–2018. Cox regression models and competing risk models were separately used to examine the association of FPG levels and long‐term fluctuation with risk of total and cause‐specific mortality.

**Results:**

Subjects who had an impaired fasting glucose or diabetes suffered a higher risk of total mortality than subjects who had a normal fasting glucose (HRs and 95% CIs: 1.17 [1.01–1.35], 1.30 [1.10–1.53], respectively). The HR for total mortality was 1.54 (95% CI: 1.29–1.84) and for cancer mortality was 1.41 (95% CI: 1.04–1.92) in the highest quartile of coefficient of variation of FPG. Trajectory analysis indicated that subjects with a significantly changed FPG suffered a higher risk of total mortality.

**Conclusion:**

According to this cohort study, we found that long‐term fluctuation of FPG was significantly associated with the risk of total and cancer mortality. Our findings suggest that long‐term fluctuation of FPG could be used as an efficient indicator for predicting the subsequent risk of mortality.

## Graphical abstract

Fasting plasma glucose variation could increase risk of cancer mortality. Controlling fasting plasma glucose fluctuations is of great health significance.

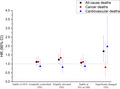

**Lay summary**

Based on this large cohort study, we found subjects who had impaired fasting glucose or diabetes mellitus suffered a higher risk of total mortality than subjects with normal fasting glucose. Cause‐specific analysis indicated that men with diabetes mellitus had a greater risk of cancer deaths than women, and no effects for cardiovascular disease among men or women. We demonstrated that variation and longitudinal changes in fasting plasma glucose were associated with higher risk of mortality. Further studies with well‐designed and longer follow‐up periods are necessary to confirm these findings.

## 1 INTRODUCTION

The level of fasting plasma glucose (FPG) is commonly used to diagnose diabetes mellitus (DM) and previous studies have shown that elevated FPG was associated with an increased risk of mortality, including cancer as well as cardiovascular disease (CVD) mortality.^1^, ^2^, ^3^, ^4^ A pooled analysis including 46,387 subjects aged 40–79 years from ten cohorts in Japan suggested that a dose‐response relationship between FPG and pancreatic cancer mortality.^4^ Some researchers reported that, mechanistically, glucose homeostasis dysfunction, which facilitated inflammation in immune microenvironment, could lead to the development and metastasis of cancer.^5^, ^6^ An umbrella review explored the relationship between type 2 DM and associated biomarkers with cancer risk, the results showed a positive association of genetically predicted FPG with squamous cell lung carcinoma.^7^ A multi‐institutional cross‐sectional study also suggested a direct positive association with an increasing risk of breast cancer recurrence and insulin resistance score which was calculated by FPG and insulin measurements.^8^

However, as a significant predictor of mortality, the legitimacy of FPG has not been well established, especially in subjects with poor glycemic control. Gujral et al. conducted a meta‐analysis and the results suggested that impaired fasting glucose (IFG) was also significantly associated with total mortality.^9^ Watt et al. reported that elevated glycated hemoglobin (HbA1c) was associated with liver fibrosis, a key risk factor for hepatocellular carcinoma, but not for elevated FPG.^10^ In addition to single‐time FPG measurement, FPG longitudinal changes may also have potential adverse effect on cancer mortality. As observed in previous studies, glucose variability could evidently predict the risk of total mortality among subjects with type 2 DM.^11^ Also, an elevated glucose variability in each year could increase cancer incidence among patients with type 2 DM, as reported by the Taichung Diabetes Study.^12^ Yet despite all that, the sample sizes of previous studies were relatively small and evidence for the association of FPG and its longitudinal changes with cancer mortality among the general population is still insufficient.

Herein, we reported the results of FPG levels at baseline and risk of cancer, as well as total and CVD mortality in a cohort study in China. Moreover, we examined the potential effects of FPG longitudinal changes on sequent risks of mortality in this population.

## 2 MATERIAL AND METHODS

### 2.1 Fasting plasma glucose assessment

Fasting blood samples, as having been fasted overnight, were drawn between 07.30 and 09.00 h. Blood samples were collected and sent to be analyzed within 4 h. A biochemical auto‐analyzer was adopted to analyze the FPG (Beckman Coulter AU5800). We divided subjects based on their FPG levels into three categories, based on the criteria issued by American Diabetes Association: diabetes mellitus (Self‐reported diabetes mellitus or FPG ≥7.0 mmol/l); impaired fasting glucose (5.6 mmol/l ≤ FPG < 7.0 mmol/l) and normal FPG (3.9 mmol/l ≤ FPG < 5.6 mmol/l).

### 2.2 Study population

We retrospectively collected the health examination information of 52,049 residents aged over 18 years with at least one FPG record from 2010 to 2014 in Yinzhou, Ningbo, a typical harbor city with sub‐provincial administrative status in the eastern part of Zhejiang Province, P. R. China. Each subject underwent a medical examination by a doctor in local village hospitals at baseline and characteristic data including sex, age, height, weight, blood pressure (BP), marital status, educational attainment, lifestyles (such as cigarette smoking, alcohol consumption and physical activity), and history of diseases of each subject was collected from the questionnaires by trained research technicians following a standardized protocol at recruitment. All questionnaires were collected for quality control and data entry (independently entered and checked by two investigators). And the data were stored in the Yinzhou Health Information System (YHIS) for subsequent extraction and use.

Height and weight were measured with no shoes for all participants, using a standard protocol. Body mass index (BMI) was calculated through dividing each subject's weight in kilograms by meters squared (kg/m^2^). BP was the average of three measurements using a mercury sphygmomanometer while seated after 10 min rest. Systolic and diastolic blood pressure were separately measured as the first and fifth Korotkoff sounds. Hypertension was defined as BP ≥140/90 mmHg. Currently smoking was defined as smoked at least one cigarette a day for a year or more. Currently drinking was defined as drinking at least 100 g of any alcohol per week. Physical activity was defined as any aerobic activity for at least 20 min.

Subjects with the following characteristics were excluded: (1) lack of accurate identity information and lost to follow‐up; (2) without marital status or educational attainment records; (3) missing records for smoking, alcohol drinking or physical activity; (4) with an extremely high or low height or weight (outliers: those higher or lower than the Mean ± 3 SDs); (5) with an extremely low FPG (outliers: FPG <3.9 mmol/l). Finally, a total of 48,173 subjects were included in the analyses. In further analyses, subjects who had at least two measurement records of FPG during 2 years were included to examine the association between FPG variability and risk of total, cancer and CVD mortality (*n* = 30,026) (Figure S1).

### 2.3 Follow up

Subjects were followed based on the Yinzhou Health Information System (YHIS), an efficient Health Information System which encompassed by residents' Health Care Recording System, Hospital Information System, Chronic Disease Recording System and Death Registration System, allowing us to obtain real‐time health events of each resident comprehensively.^13^

Death status of each subject was identified by official death certificates at Yinzhou Center for Disease Control and Prevention (Yinzhou CDC). The primary causes of death were identified by the International Classification of Disease, 10th Revision (ICD‐10) (C00–C97 for cancer deaths and I00–I99 for CVD deaths). Our study began at 2010 and subjects were recruited into our cohort in succession and followed up until Dec. 31, 2018. In further analyses, the initial date of follow‐up was defined according to the most recent examination of FPG. Subjects were censored at their dates of deaths, last known follow‐up dates or Dec. 31, 2018, whichever occurred first.

### 2.4 Statistical analysis

Differences among three FPG level groups were separately tested by using Chi‐squared tests (categorical variables) and Analysis of Variance (ANOVA) (continuous variables). We tested the assumption of proportional hazard for each variable in our models and the results are presented in Table S1. In the primary analyses, Cox regression model was used to estimate hazard ratios (HRs) and 95% confidence intervals (95% CIs) to evaluate the association of FPG levels with risk of all‐cause deaths. Variables (including physical activity and history of hypertension) that did not satisfy the hypothesis test of proportional hazard assumption were stratified in the analysis. Competing risk models were used to estimate the association of FPG with risk of cause‐specific deaths. Potential confounding factors included age at baseline (continuous variable), sex (dummy variable), BMI (rank variable), marital status (dummy variable), educational attainment (rank variable), cigarette smoking (dummy variable), alcohol drinking (dummy variable), and physical activity (dummy variable).

We also performed sensitivity analyses by excluding subjects who were followed up less than three years and subjects with chronic disease including cancer and CVD at baseline. Subgroup analyses were conducted stratified by age at baseline (<60 or ≥60 years) and sex (men or women). In further analyses, we also used Cox regression model to evaluate the association of long‐term fluctuation of FPG with risk of all‐cause deaths. Competing risk models were used to estimate long‐term fluctuation of FPG with risk of cause‐specific deaths. Two measures were calculated as indicators for long‐term fluctuations in FPG in our study: the coefficient of variation of FPG (FPG‐CV) and the trajectory pattern of FPG. FPG‐CV was calculated by all FPG level records during the whole follow‐up, and the trajectory pattern of FPG was measured over months by baseline and the latest FPG levels. According to the FPG‐CV, we divided the participants into four groups by quartiles and the lowest quartile was considered as a reference. Moreover, in further analyses, we also divided subjects into five different groups to explore the relationship between longitudinal change in FPG levels and risk of mortality: (1) stable in NFG: NFG to NFG; (2) gradually controlled FPG: IFG to NFG or DM to IFG; (3) slightly elevated FPG: NFG to IFG or IFG to DM; (4) stable in IFG or DM: IFG to IFG or DM to DM; (5) significantly changed FPG: NFG to DM or DM to NFG. Stable normal FPG level was used as the reference. We considered two‐sided *p*‐value < 0.05 as statistically significant and all analyses were conducted by using R software (version 3.6.1).

## 3 RESULTS

Information about age, weight, height, lifestyle factors, and vital status was available for 48,173 participants (including 20,650 men and 27,523 women) with different FPG levels at baseline (Table 1). During the 7.4 years of median follow‐up, totally 1836 death cases were identified in this study, including 683 deaths from cancer and 558 deaths from CVD. As shown in Table 1, all characteristics varied across different FPG groups, except for disease status of cancer at baseline.

**TABLE 1:** Baseline characteristics according to different clinical categories of fasting plasma glucose

Characteristics	Total (*N* = 48,173)	Normal fasting glucose (*N* = 34,585)	Impaired fasting glucose (*N* = 10,602)	Diabetes mellitus (*N* = 2986)	*p*‐value^d^
Women (*n*, %)	27,523 (57.1)	19,819 (57.3)	5870 (55.4)	1834 (61.4)	<0.001
Age, mean (SD), years	58.0 (11.6)	57.1 (11.8)	60.1 (10.8)	62.1 (10.4)	<0.001
Height, mean (SD), cm	161.8 (7.3)	161.9 (7.2)	161.7 (7.5)	160.5 (7.5)	<0.001
Weight, mean (SD), kg	60.5 (8.2)	60.2 (8.0)	61.3 (8.5)	61.4 (9.0)	<0.001
Body mass index (kg/m^2^)	23.1 (2.6)	22.9 (2.5)	23.5 (2.8)	23.8 (2.9)	<0.001
Marriage (*n*, %)					<0.001
Married	42,753 (88.7)	30,615 (88.5)	9507 (89.7)	2631 (88.1)	
Single	2523 (5.2)	2072 (6.0)	365 (3.4)	86 (2.9)	
Divorced or widowed	2897 (6.1)	1898 (5.5)	730 (6.9)	269 (9.0)	
Education (*n*, %)					<0.001
College or more	138 (0.3)	125 (0.4)	9 (0.1)	4 (0.1)	
Middle or high school graduate	16,333 (33.9)	12,723 (36.8)	2914 (27.5)	696 (23.3)	
1–6 years education	31,702 (65.8)	21,737 (62.8)	7679 (72.4)	2286 (76.6)	
Cigarette smoking^a^ (*n*, %)					<0.001
Never smoking	35,273 (73.2)	25,302 (73.2)	7737 (73.0)	2234 (74.8)	
Formerly smoking	3385 (7.0)	2193 (6.3)	890 (8.4)	302 (10.1)	
Current smoking	9515 (19.8)	7090 (20.5)	1975 (18.6)	450 (15.1)	
Alcohol consumption^b^ (*n*, %)					0.031
Often drinking	7210 (15.0)	5000 (14.5)	1732 (16.3)	478 (16.0)	
Occasionally drinking	3049 (6.3)	2228 (6.4)	648 (6.1)	173 (5.8)	
Never drinking	37,914 (78.7)	27,357 (79.1)	8222 (77.6)	2335 (78.2)	
Physical activity (/week)^c^ (*n*, %)					<0.001
More than 4 times	22,496 (46.7)	16,453 (47.6)	4735 (44.7)	1308 (43.8)	
1–4 times	14,088 (29.2)	9714 (28.1)	3356 (31.7)	1018 (34.1)	
Less than 1 time	11,589 (24.1)	8418 (24.3)	2511 (23.7)	660 (22.1)	
History of cancer (*n*, %)	1093 (2.6)	737 (2.1)	284 (2.7)	72 (2.4)	0.004
History of hypertension (*n*, %)	21,485 (52.4)	13,713 (39.7)	5851 (55.2)	1921 (64.3)	<0.001
History of CVD (*n*, %)	891 (2.2)	533 (1.5)	256 (2.4)	102 (3.4)	<0.001

Abbreviations: CVD, cardiovascular disease; SD, standard deviation.

^a^ Currently smoking was defined as smoking at least one cigarette a day for 1 year or more.

^b^ Currently drinking was defined as consuming at least 100 g of any alcohol a week.

^c^ Physical activity was defined as any aerobic exercise for at least 20 min.

^d^ Student's *t*‐test and *χ* ^2^ test were used to examine the differences between men and women in basic characteristics.

The HRs and 95% CIs for the associations of FPG levels with total and cause‐specific mortality are presented in Table 2. In the primary analyses, we found that subjects with IFG or DM had a higher risk of total mortality than subjects with NFG, and the corresponding HRs were 1.14 (1.03–1.27) and 1.33 (1.12–1.57), respectively. Cause‐specific mortality analyses suggested that compared with subjects with NFG, subjects with DM had a significantly increased risk of cancer mortality by 37% (95% CI: 3%–81%), but not for CVD mortality. Similar results were observed for total mortality in sensitivity analyses by excluding subjects who were followed up less than three years. The corresponding HRs for cancer deaths were 1.67 (95% CI: 1.18–2.36) among subjects with DM, but no association for CVD mortality. Results from the subgroup analyses stratified by age at baseline (<60 or ≥60 years) and sex (men or women) were separately shown in Figures S2 and S3. The younger subjects and men with DM had a higher risk of total mortality. A stronger association was observed for DM and risk of all‐cause deaths among younger subjects (HR = 1.89, 95% CI: 1.03–3.49) and men (HR = 1.46, 95% CI: 1.16–1.84). No associations were observed for women.

**TABLE 2:** Hazard ratios (HRs) for the association of FPG levels at baseline with risk of all‐cause, cardiovascular and cancer mortality in primary and sensitivity analyses

	Deaths from any cause^a^		Cancer deaths^a^		Cardiovascular disease deaths^a^	
	HR and 95% CI	*p*	HR and 95% CI	*p*	HR and 95% CI	*p*
Primary analyses						
NFG	1.00 [Ref]		1.00 [Ref]		1.00 [Ref]	
IFG	1.14 (1.03–1.27)	0.016	1.16 (0.98–1.38)	0.100	1.00 (0.82–1.22)	0.970
DM	1.33 (1.12–1.57)	0.001	1.37 (1.03–1.81)	0.029	1.07 (0.79–1.44)	0.680
Sensitivity analyses I^b^						
NFG	1.00 [Ref]		1.00 [Ref]		1.00 [Ref]	
IFG	1.11 (0.97–1.28)	0.132	1.07 (0.84–1.36)	0.600	0.94 (0.73–1.23)	0.660
DM	1.40 (1.13–1.74)	0.002	1.67 (1.18–2.36)	0.004	1.13 (0.76–1.67)	0.540
Sensitivity analyses II^c^						
NFG	1.00 [Ref]		1.00 [Ref]		1.00 [Ref]	
IFG	1.16 (1.04–1.29)	0.006	1.18 (1.00–1.41)	0.055	1.03 (0.85–1.26)	0.760
DM	1.31 (1.11–1.55)	0.001	1.26 (0.96–1.67)	0.098	1.13 (0.83–1.52)	0.440

Abbreviations: 95% CI, 95% confidence interval; DM, diabetes mellitus; HR, hazard ratio; IFG, impaired fasting glucose; NFG, normal fasting glucose.

^a^ Multivariable adjustment for age, sex, marriage, education, BMI, smoking, alcohol drinking, exercise, and chronic disease (including tumor, hypertension, and cardiovascular disease).

^b^ Sensitivity analyses I by excluding subjects who were followed up less than three years.

^c^ Sensitivity analyses II by excluding subjects with chronic disease including cancer or CVD at baseline.

**FIGURE 1:**
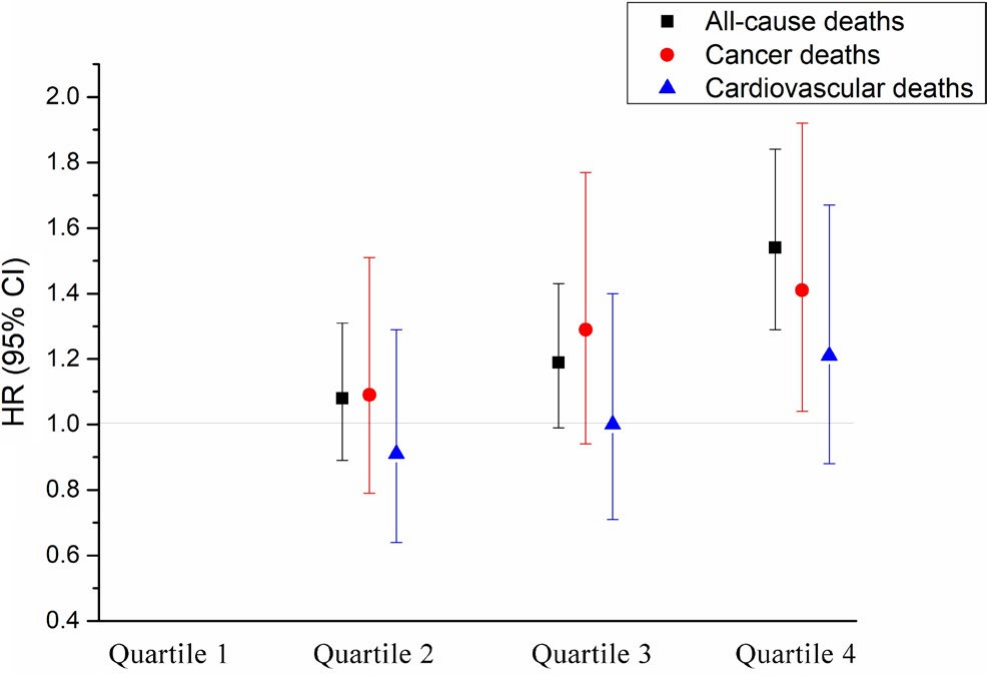
Hazard ratios (HRs) for risk of total, cardiovascular disease and cancer mortality according to the coefficient of variation of fasting plasma glucose (FPG‐CV)

**FIGURE 2:**
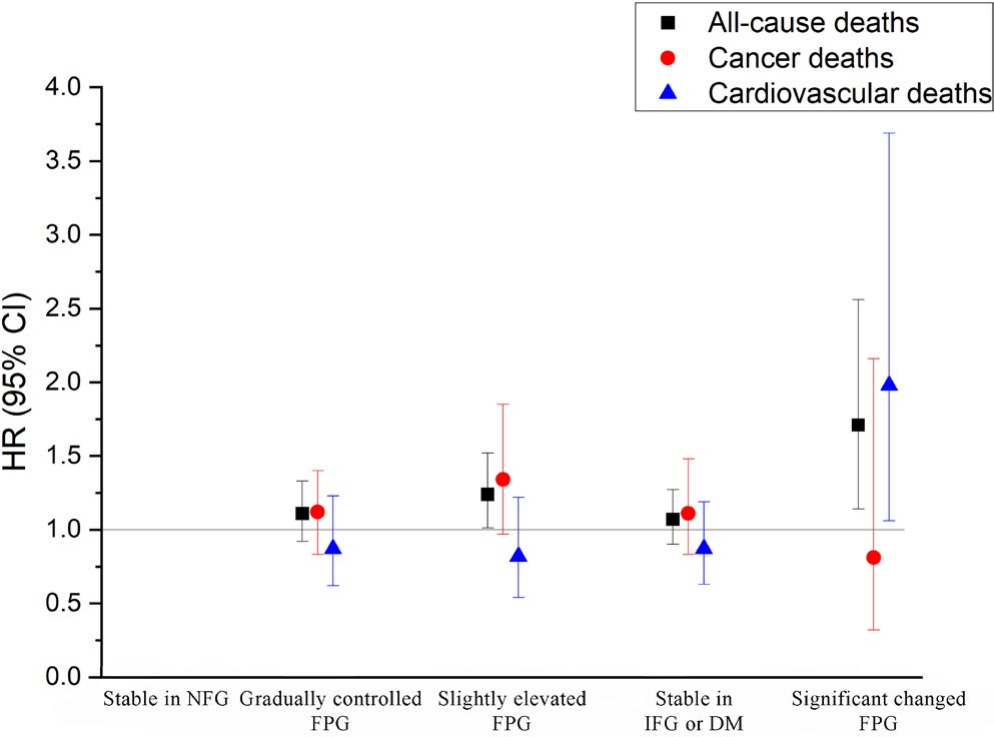
Hazard ratios (HRs) for risk of all‐cause, cardiovascular disease and cancer mortality according to fasting plasma glucose (FPG) trajectory patterns

In further analyses, we included a total of 30,026 participants with at least two measurement records of FPG levels, and 973 deaths were identified in this study (including 371 cancer deaths and 299 CVD deaths). Figure 1 presents the associations of FPG‐CV with the risks of total and cause‐specific mortality. Compared with the lowest quartile of FPG‐CV, subjects in the highest quartile of FPG‐CV had a significantly increased risk of total and cancer mortality by 54% (95% CI: 29%–84%) and 41% (4%–81%), respectively. Figure 2 presents the associations of FPG trajectory patterns with the risks of total and cause‐specific mortality. Compared with subjects in reference group (stable in NFG), subjects who were stable in IFG or DM suffered a significantly higher risk of total mortality (HR = 1.24, 95% CI: 1.01–1.52). A stronger association was observed for total mortality among participants with a significantly changed FPG (HR = 1.71, 95% CI: 1.14–2.56). However, no effects were observed in any FPG trajectory patterns for cancer mortality.

## 4 DISCUSSION

In this study, we examined the association of FPG at baseline and its longitudinal changes with the risk of mortality in a Chinese population. Overall, during the 345,922.4 person‐years of follow‐up, we found that IFG and DM were consistently associated with a higher risk of total mortality. Also, subjects with DM suffered a higher risk of cancer mortality. In further analyses, results from Cox regression model and competing risk models suggested that subjects in the highest quartile of FPG‐CV group had a significantly higher risk of total and cancer mortality than subjects in the lowest quartile. Trajectory analyses indicated that subjects who were stable in IFG or DM or had a substantially changed FPG suffered a higher risk of total mortality than subjects who were stable in NFG.

Several epidemiology studies have reported that increased FPG is a crucial risk factor for cancer occurrence, especially for pancreatic, colorectal, and breast cancers.^4^, ^14^, ^15^, ^16^ Increased FPG is also considered as a potential risk factor for CVD. Previous studies suggested that increased FPG can lead to a variety of CVD complications, such as heart failure, coronary artery disease, and heart hypertrophy.^17^, ^18^, ^19^, ^20^, ^21^ An early meta‐analysis included 20 studies reported that the progressive association of FPG levels with the risk of CVD extended below the diabetic threshold.^18^ Laukkanen et al. in 2013 reported that IFG was significantly associated with the risk of sudden cardiac deaths (SCDs) among patients with type 2 diabetes, and every 1 mmol/l increment in FPG was in relation to an increase of 10% in the risk of SCDs.^22^ However, one limitation existed that the authors did not conduct a competing risk analysis in their study.

In recent years, glucose variability has also been used as an important indicator for serious health outcomes.^11^, ^12^, ^23^, ^24^, ^25^, ^26^, ^27^ Nonetheless, the effects of glucose variability on mortality was examined mostly among diabetic patients and limited studies can be available in the general population. A recent research conducted in the general population revealed that the elevated visit‐to‐visit variability (VVV) of FPG was in relation to an increased risk of total mortality.^28^ This finding was confirmed in a South Korean cohort study, and the researchers also found the relationship of increased glucose variability on the development of type 2 diabetes.^29^

Our findings for the relationship of FPG at baseline and variability on the risk of mortality basically align with other studies on this topic. Some caution is necessary when interpreting our results. Age of the onset of DM may affect the risk of mortality among residents with diabetes.^30^, ^31^ A recent data from a large cohort including 90% of Australian type 2 diabetes showed that a 10‐year earlier diagnosis was significantly associated with a 1.2–1.3 times increased risk of total mortality.^32^ In our study, IFG and diabetes seemed to be significantly associated with a higher risk of mortality in younger participants (<60 years) instead of in older ones (≥60 years), though this difference was not statistically significant. One possible explanation is that younger‐onset diabetes may result in longer duration of hyperglycemia and earlier CVD mortality.^30^, ^32^ Subgroup analyses by sex did not alter our results in general and FPG levels were more strongly associated with mortality risk in men than in women. It has been reported that premenopausal women are at a lower risk of CVD mortality, because of the protective effect of estrogen on cardiovascular tissues. In addition, this vascular protective effect of estrogen faded significantly for diabetic women.^33^ Thus, a longer‐term follow‐up is necessary to estimate the effects of FPG on mortality among women.

One main advantage of this cohort study is the relatively large sample size. Based on the YHIS, we were able to recruit a total of 48,173 residents and obtain the real‐time health events of each subject. The applicability of each participant's medical records allowed us to further evaluate the effects of FPG longitudinal changes on the risk of total or cause‐specific mortality. Also, we adjusted for a number of potential confounding factors including age at baseline, sex, BMI, marital status, education, smoking, drinking, physical activity, and chronic disease at baseline.

Nevertheless, our study also has several limitations. Firstly, the median follow‐up time of this study was relatively insufficient (only 7.4 years). The insufficient size of death events (especially for CVD deaths) could limit the study power and affect our findings. Secondly, glycated hemoglobin, as one of the crucial independent risk factors of CVD events, was not available for all participants at baseline, which may affect our results. Thirdly, in further analyses, we excluded participants who had only one measurement record of FPG. Difference in general characteristics between one measurement and more than two measurements was not obvious, indicating that the influence of the number of measurements on the results was weak. Besides, the trajectory pattern of FPG was measured only by baseline and the latest FPG levels and more detailed changes in FPG levels were not included in the study due to differences in the number of FPG records for each subject. Finally, some caution is still essential when applying our findings and for that we only included subjects in Yinzhou.

## 5 CONCLUSIONS

In summary, in this large sample‐size cohort study, subjects with IFG or DM suffered a significantly higher risk of total mortality than subjects with NFG. Cause‐specific analysis indicated that DM was significantly associated with cancer mortality for men, but not for women, and no effects for CVD among men or women. We demonstrated that variation and longitudinal changes in FPG were associated with a more adverse health effect. Further studies with well‐designed and longer follow‐up periods are necessary to confirm these findings.

## ETHICS STATEMENT

Our study has been filed with the Ethics Review Committee of Yinzhou CDC and has been reviewed and approved by the Ethics Review Committee of Yinzhou CDC, the IRB number is 2020‐01. And the requirement for informed consent was waived due to the retrospective design.

## DATA AVAILABILITY STATEMENT

The processed data required to reproduce these findings cannot be shared at this time as the data also form part of an ongoing study.

## CONFLICT OF INTEREST

The authors declare that they have no known competing financial interests or personal relationships that could have appeared to influence the work reported in this paper.

## AUTHOR CONTRIBUTIONS

**MY Wu:** Conceptualization, Resources, Writing—review & editing, Methodology, Software. **JM Lu:** Resources, Validation. **ZM Yang:** Resources, Validation. **P Shen:** Data curation. **ZB Yu:** Resources. **ML Tang:** Supervision. **MJ Jin:** Supervision. **HB Lin:** Data curation. **K Chen:** Supervision. **JB Wang:** Conceptualization, Methodology, Writing—review & editing, Funding acquisition.

## Supporting information

Supplementary Material
