# Supplementary material for: Longitudinal changes in fasting plasma glucose are associated with risk of cancer mortality: A Chinese cohort study
Source: Cancer Med. 2021 Jun 21;10(15):5321–8. doi: 10.1002/cam4.4070 (PMC8335834; doi:10.1002/cam4.4070)
Supplement: Supplementary file 1 — Supplementary Material [file CAM4-10-5321-s001.docx]

**Supplementary materials**


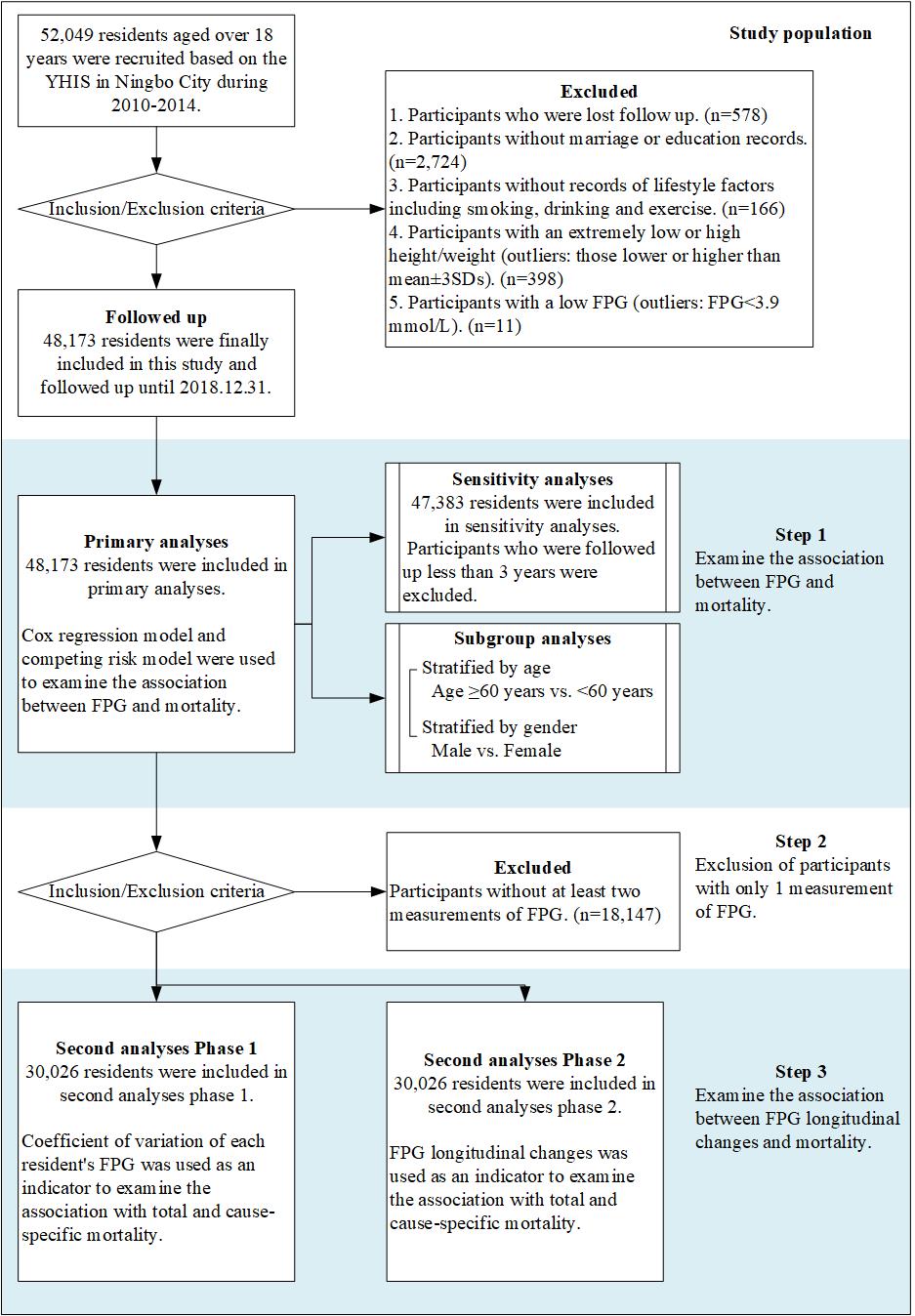


**Figure S1. Study population**

**Table S1. Results of the test for proportional hazards assumption**

| **Characteristics** | **Test for proportional hazards** | | |
| --- | --- | --- | --- |
|  | Wald $\chi^{2}$ | Df | *P* |
| Sex | 1.690 | 1 | 0.139 |
| Age | 0.014 | 1 | 0.606 |
| Body Mass Index | 0.084 | 1 | 0.512 |
| Marriage | 0.023 | 2 | 0.965 |
| Education | 2.250 | 2 | 0.419 |
| Cigarette Smoking | 4.200 | 2 | 0.052 |
| Alcohol Consumption | 2.681 | 2 | 0.239 |
| Physical Activity(/week) | 8.851 | 2 | 0.012 |
| History of cancer | 1.655 | 1 | 0.914 |
| History of hypertension | 9.298 | 1 | 0.002 |
| History of CVD | 2.759 | 1 | 0.097 |
| FPG | 4.582 | 2 | 0.101 |

Abbreviations: Df, degree of freedom; CVD, Cardiovascular Disease; FPG, fasting plasma glucose.


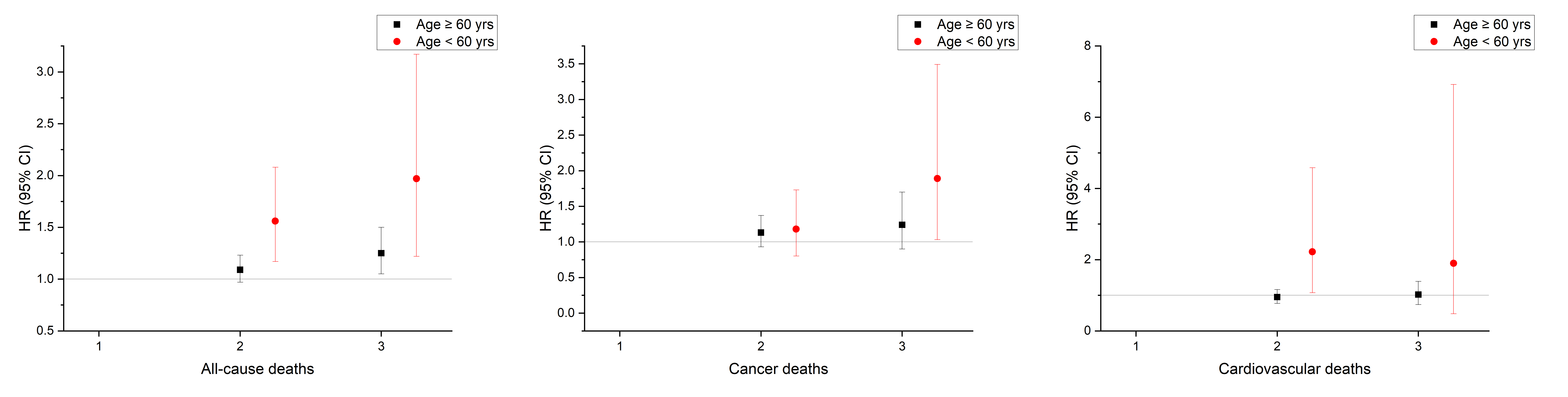


**Figure S2. Subgroup analyses for Hazard ratios (HRs) for risk of all-cause, cardiovascular and cancer mortality according to FPG levels stratified by age**


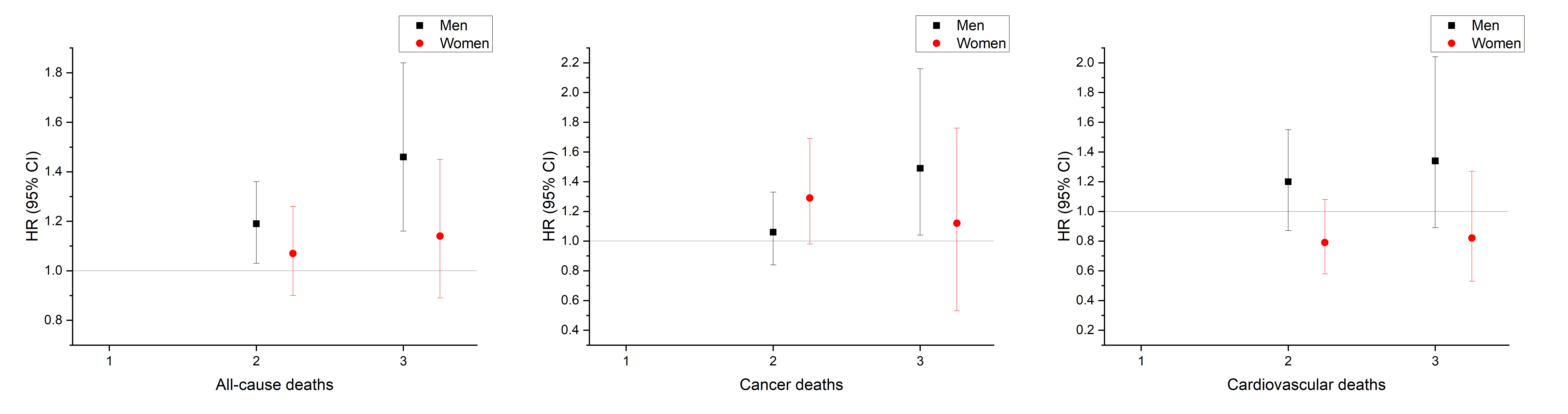


**Figure S3. Subgroup analyses for Hazard ratios (HRs) for risk of all-cause, cardiovascular and cancer mortality according to FPG levels stratified by sex**
